# Biochemical and clinical characterization of metabolic phenotypes: a cross-sectional study from Maracaibo city, Venezuela

**DOI:** 10.12688/f1000research.13897.1

**Published:** 2018-02-27

**Authors:** Valmore Bermudez, Joselyn Rojas, Juan Salazar, Maria Sofia Martinez, Luis Carlos Olivar, Maria Jose Calvo, Andres Mindiola, Roberto Añez, Sandra Wilches-Duran, Marcos Cerda, Modesto Graterol, Rosemily Graterol, Juan Diego Hernandez, Carlos Garicano, Manuel Velasco

**Affiliations:** 1 Grupo de Investigación Altos Estudios de Frontera (ALEF), Universidad Simón Bolívar, Cucuta, Colombia; 2 Endocrine and Metabolic Diseases Research Center, The University of Zulia, Maracaibo, Venezuela; 3 Pulmonary and Critical Care Medicine Department, Brigham and Women’s Hospital, Harvard Medical School, Boston, MA, USA; 4 Geriatric Research Education and Clinical Center (GRECC), U.S Department of Veterans Affairs, Miami, FL, USA; 5 Department of Pharmacology, “JM Vargas” Medical School, Central University of Venezuela, Caracas, Venezuela

**Keywords:** Metabolic phenotypes, two-step cluster, metabolically unhealthy lean, metabolically healthy obese, coronary risk

## Abstract

**Background: **In 1980, Reuben Andresen observed that in certain individuals, obesity did not increase mortality, introducing an atypical phenotype called “healthy obese”. Other studies reported that 10-15 % of lean individuals presented insulin resistance, hyperglycemia and dyslipidemia. The objective of this study was to evaluate biochemical and clinical characteristics of metabolic phenotypes in Maracaibo city.

**Methods:** A descriptive, cross-sectional study with a randomized multistage sampling was performed including 1226 non diabetic individuals from both sexes. For phenotype definition, the subjects were first classified according to their BMI into Normal-Weight, Overweight and Obese; then divided in metabolically healthy and unhealthy using a two-step analysis cluster. To evaluate the relationship with coronary risk, a multiple logistic regression model was performed.

**Results: **In the studied population, 5.2% (n=64) corresponded to unhealthy lean subjects, and 17.4% (n=217) to healthy obese subjects. Metabolically unhealthy normal-weight (MUNW) phenotype was found in males in 53.3% in contrast to 51.3% of metabolically unhealthy obese (MUO) phenotype found in females. An association between metabolically unhealthy phenotypes and a higher risk of a coronary event was found, especially for obese individuals (MHO: OR=1.85 CI95%: 1.11-3.09; p=0.02 and MUO: OR=2.09 CI95%: 1.34-3.28; p<0.01).

**Conclusion:** Individuals with atypical metabolic phenotypes exist in Maracaibo city. Related factors may include insulin resistance, basal glucose levels, and triglycerides levels. Lastly, cardiovascular risk exhibited by healthy obese individuals should be classified in categories of major coronary risk related to lean subjects.

## Introduction

Obesity is considered an entity with major morbi-mortality in the world since the end of the 20th century ^1^. Multiples studies have shown its role as an independent risk factor for various cardiometabolic disorders such as hypertension (HTN), dyslipidemias, Type 2 Diabetes Mellitus (T2DM) and cardiovascular disease (CVD) ^2^. For this reason, the actual clinical practice catalogues an obese patient as an “unhealthy” patient and a lean patient is considered “healthy”.

In spite of this, in 1980, Reuben Andresen discovered that in certain groups of individuals the obesity was not a mortality increasing factor, introducing the subtype “Healthy Obese” ^3^. Around 20 years later, Ferranini *et al.* observed that a group of certain obese nondiabetic non-hypertensive subjects presented low insulin resistance (IR) prevalence, suggesting that this subtype must have a different risk of having T2DM and CVD from the IR obese; also suggesting a different management for them ^4^.

Furthermore, in 1975, Bernstein *et al.* observed that 11 normal-weight men with type IV or V dyslipidemia presented higher serum glucose levels; and also carried bigger sized adipocytes with respect to their healthy counterparts ^5^. Years later, Ruderman et al. introduced the “Metabolically Unhealthy Normal-Weight” phenotype attributed to lean individuals with metabolic alterations associated to obesity ^6^.

The importance of these atypical metabolic phenotypes lies in the fact that their diagnosis may be challenging for clinicians delaying their detection. Because of this, in recent years, multiple studies have been dedicated to the research of accurate clinical, biochemical, and genetic elements capable to detect these atypical metabolic states, and their evolution.

In this sense, these phenotypes determinants and frequencies have not been deeply researched in Latin-American populations ^7^. The objective of this study is to characterize, from a clinical-biological point of view, the metabolic phenotypes in the population from Maracaibo city, Venezuela.

## Materials and methods

### Population selection

The Maracaibo City Metabolic Syndrome Prevalence Study (MMSPS) is a cross-sectional study whose purpose is to detect metabolic syndrome and cardiovascular disease risk factors in the adult population from Maracaibo, the second largest city of Venezuela, with approximately 2,500,000 inhabitants, during the period May 2007 – December 2009. The original study included a total of 2230 individuals of both genders, aged between 18–85 years old, and the study protocol was previously reported ^8^. This sub-analysis excluded those individuals with no measurements of serum insulin levels. Patients with past history of diabetes were also excluded because their disease control, evolution and pharmacological treatments would affect the variables in the study.

**Figure 1.**
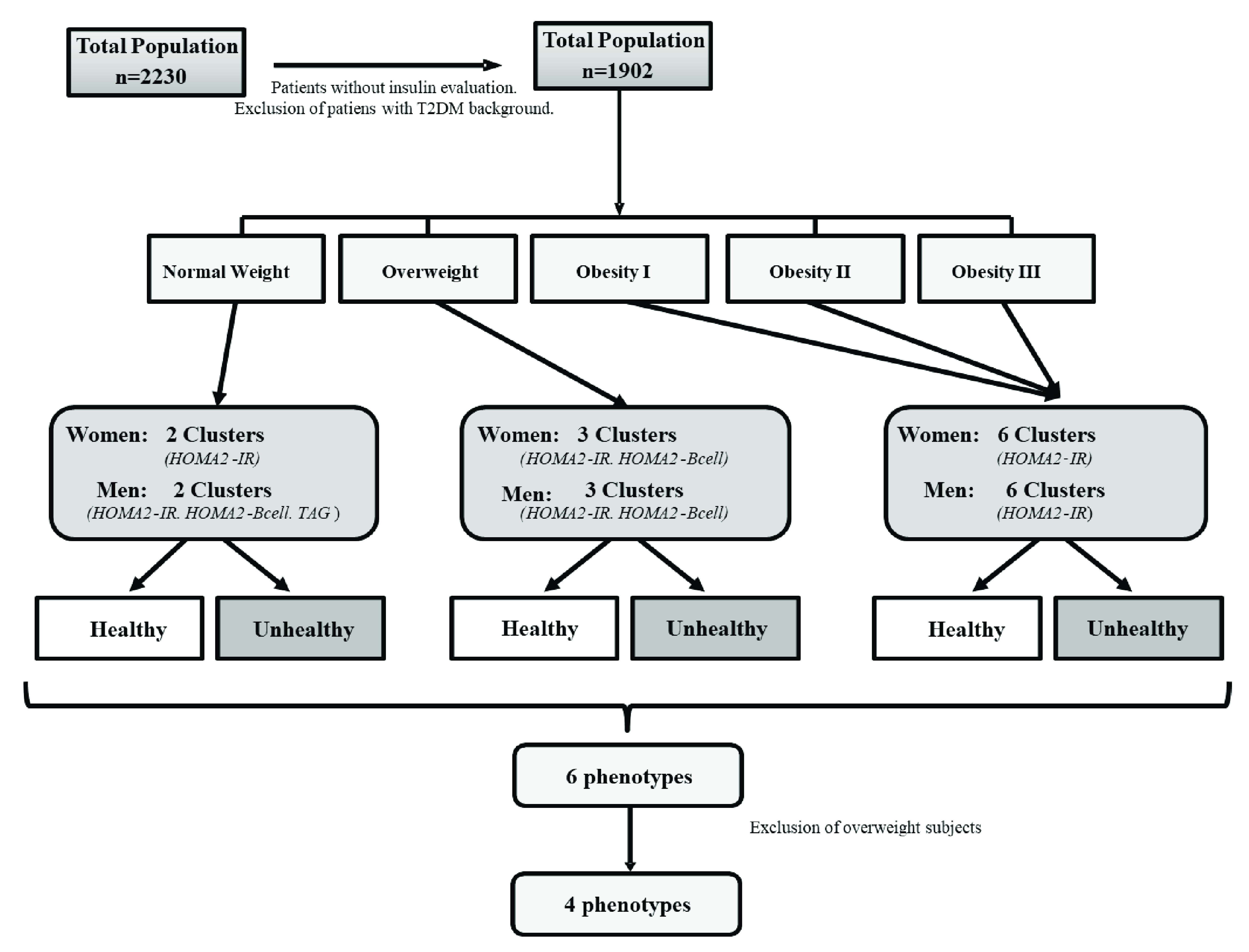
Patient selection diagram. Maracaibo city, Venezuela. During simple selection, subjects with no measurements of serum insulin levels and patients with past history of diabetes were excluded. These subjects were categorized into six groups, first according to their BMI and second to their healthy/unhealthy definition, using two-step cluster analysis.

These subjects were categorized into six groups, first according to their Body Mass Index (BMI) (normal-weight, overweight and obese) and second, to their healthy/unhealthy definition. This categorization was made using the protocol from two-step cluster analysis published previously ^9^. The metabolic variables were chosen as possible metabolic predictors based on their physiological function and biological plausibility. These variables were: mean arterial pressure (MAP), triglycerides (TAG), total cholesterol, HDL-C, HOMA2-IR, HOMA2-βcell, HOMA2-S, fasting blood glucose, non-HDL-C cholesterol, TAG/HDL-C ratio, and high-sensitivity C-Reactive Protein (hs-CRP) levels; waist circumference (WC) was excluded and was assessed as a dependent variable. The predictive strength of these variables was analyzed in accordance to cluster ability and quality, ranging from 0.0 to 1.0. The most appropriate predictive variables selected for each group were: (a) HOMA2-IR and HOMA2-βcell for normal-weight women; (b) HOMA2-IR, HOMA2-βcell and TAG for normal-weight men; (c) HOMA2-IR and HOMA2-βcell for overweight women; (d) HOMA2-IR, HOMA2-βcell, and TAG for overweight men; and (e) HOMA2-IR for male and female obese patients. The two-step cluster analysis with SPSS was conducted in two phases: during the first step (called “precluster”), the subjects were divided into several small subclusters. Then, the obtained subclusters were grouped in preferred number of clusters; if the desired number of clusters was unknown, the SPSS two-step cluster component would find the proper number of clusters automatically. Once the program analyzed the subclusters with the characteristics of each BMI category (as described previously), the subjects were categorized into 6 phenotypes: healthy normal-weight (HNW), metabolically unhealthy normal-weight (MUNW), healthy and metabolically disturbed overweight, metabolically unhealthy obese (MUO), and metabolically healthy obese (MHO). Overweight subjects were excluded from this secondary analysis since they represent a non-conventional group outside the metabolic phenotypes and require separate analysis. The final sample included 1226 subjects ( Figure 1).

### Clinical evaluation

Data was collected through completion of a full clinical record carried out by trained personnel, which included interrogation regarding ethnic origin and socioeconomic status by the Graffar scale according to Méndez-Castellano ^10^. The assessment of blood pressure was done by applying the auscultatory technique, and HTN classification was made using the criteria proposed in the VII Joint National Committee on Prevention, Detection, Evaluation, and Treatment of High Blood Pressure ^11^.

For Anthropometric Analysis, an electrical bioelectric scale was used to obtain weight (Tanita, TBF-310 GS Body Composition Analyzer, Tokyo – Japan). Height was measured using a calibrated metric measurement tape, with the subject standing up barefoot. BMI formula (weight/height ^2^) was applied, expressing the results as kg/m ^2^. Obesity was classified applying the WHO criteria ^12^ based on the BMI value. Finally, WC was measured using calibrated measuring tape in accordance to the anatomical landmarks proposed by the USA National Institutes of Health protocol ^13^.

***Physical activity***. Physical activity (PA) was assessed with the International Physical Activity Questionnaire (IPAQ). For statistical analysis, PA was evaluated in 4 domains: occupational, household, transport, and leisure. In each of these domains, subjects were categorized as follows: (a) inactive, MET/week = 0, or (b) active, MET/week > 0. The latter were then subcategorized by gender-specific MET/week quintiles in each domain ( Table 1), which were published previously ^14^.

**Table 1.** Gender-specific MET quintiles for each domain of physical activity. Maracaibo city, Venezuela.

MET Quintiles *	Females		Males	
Work Domain	Lower Limit	Upper Limit	Lower Limit	Upper Limit
Very Low	33.00	385.99	33.00	714.99
Low	386.00	1201.49	715.00	2042.09
Moderate	1201.50	2751.59	2042.10	3578.39
High	2751.60	4546.79	3578.40	6495.59
Very High	4546.80		6495.60	
Transport Domain	Lower Limit	Upper Limit	Lower Limit	Upper Limit
Very Low	33.00	131.99	33.00	164.99
Low	132.00	230.99	165.00	257.49
Moderate	231.50	346.49	247.50	521.09
High	346.50	700.79	521.10	1385.99
Very High	700.80		1386.00	
Household Domain	Lower Limit	Upper Limit	Lower Limit	Upper Limit
Very Low	30.00	539.99	30.00	269.99
Low	540.00	1139.99	270.00	629.99
Moderate	1140.00	1919.99	630.00	1084.99
High	1920.00	3779.99	1085.00	2429.99
Very High	3780.00		2430.00	
Leisure Domain	Lower Limit	Upper Limit	Lower Limit	Upper Limit
Very Low	33.00	230.99	33.00	296.99
Low	321.00	445.49	297.00	791.99
Moderate	445.50	742.49	792.00	1532.39
High	742.50	1798.79	1532.40	2879.99
Very High	1798.80		2880.00	

*Obtained from IPAQ scoring. Subjects with 0 MET were excluded from quintiles and classified separately as Inactive.

### Biochemical analyses

Fasting levels of glucose, cholesterol, TAG, HDL-C, and hs-CRP were assessed in our clinical laboratory using an automatized computer analyzer (Human Gesellschaft fur Biochemica und Diagnostica mbH). LDL-C and VLDL-C levels were calculated applying the Friedewald formulas ^15^. When TAG were over 400 mg/dL measurement was done using lipoprotein electrophoresis and optical densitometry (BioRad GS-800 densitometer, USA). Lipoprotein (a) [Lp(a)] was estimated through the latex turbidimetric method, Human Gesellschaft für Biochemica and Diagnostica, Germany. Likewise, serum hs-CRP levels were quantified employing immunoturbidimetric essays (Human Gesellschaft für Biochemica and Diagnostica MBH). Insulin was determined using an ultrasensitive ELISA method (DRG Instruments GmbH, Germany, International DRG Division, Inc.). For the evaluation of insulin resistance (IR), the HOMA2-IR model proposed by Levy *et al.* was utilized ^16^ determined through the HOMA-Calculator v2.2.2 program. Visceral Adiposity Index (VAI) calculation was performed with the gender-specific equations proposed by Amato *et al.* ^17^. The Metabolic Syndrome (MS) diagnosis was done using the Harmonizing-2009 consensus criteria ^18^.

### Calibration of the Framingham-Wilson equation and coronary risk categorization for the population of Maracaibo city

For proper equation calibration, the constants in the formula regarding major cumulative coronary events (lethal and non-lethal myocardial infarction, symptomatic and no symptomatic angina) were substituted with the local statistics obtained from the Vital Statistics Yearbook of the State of Zulia from 2008, where the morbidity and mortality for cardiovascular diseases is registered, the calibration process has been detailed previously ^19^. The coronary risk was classified in 2 categories: <5% in 10 years, and ≥5% in 10 years.

### Statistical analysis

Normal distribution of continuous variables was assessed using Geary’s test; for normally distributed variables, the results were expressed as arithmetic mean ± SD (standard deviation). Variables without normal distribution were logarithmically transformed, and normal distribution subsequently corroborated. When normalization could not be achieved, these variables were expressed as medians (25 ^th^ percentile–75 ^th^ percentile). Student’s –test/One-way ANOVA or Mann-Whitney/Kruskal Wallis’s tests were applied to evaluate differences between means or medians, respectively. Qualitative variables were expressed as absolute and relative frequencies, assessed through the χ ^2^ test and the Z test for Proportions.

A logistic regression model was constructed with coronary risk as dependent variable and independent variables: gender, age groups, ethnicity, socioeconomic status, smoking habit, physical activity in leisure time, elevated TAG, and metabolic phenotypes. Database construction and statistical analysis were done using the Statistical Package for the Social Sciences (SPSS) v22 for Windows (IBM Inc., Chicago, IL), results were considered statistically significant when *p*<0.05.

## Results

### Population general characteristics

**Figure 2.**
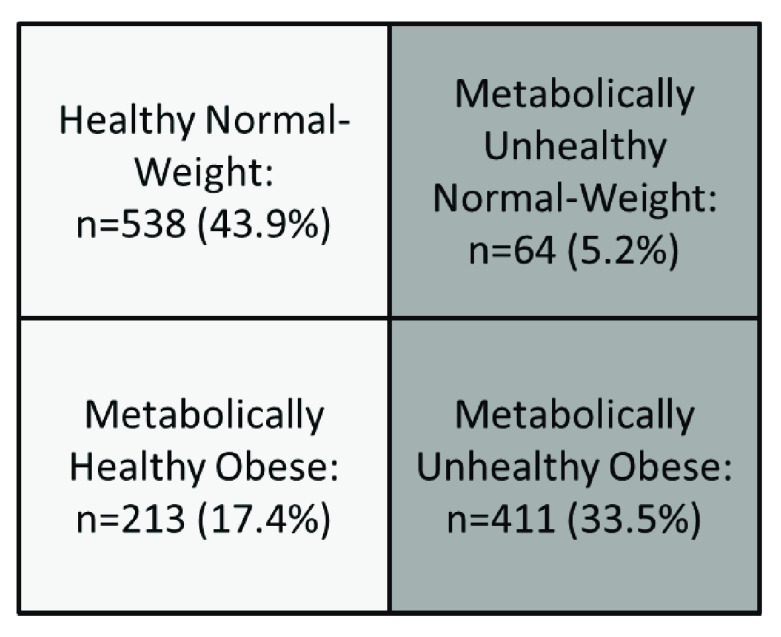
Distribution of individuals according to metabolic phenotypes. Maracaibo city, Venezuela. For this sub-analysis overweight subjects were excluded, evaluating only the typical obesity phenotypes with 4 groups.

A total of 1226 individuals were studied, 55.1% (n=676) corresponded to females and 44.9% (n=550) to males. The mean age (years) of the general population was 37.94±14.99. Subjects distribution according to their metabolic phenotype is shown in Figure 2 where the 5.2% (n=64) of the individuals were classified as MUNW, and 17.4% (n=213) as MHO, representing 34.13% from the total of obese subjects, while sociodemographic and metabolic characteristics from the studied simple are shown in Table 2.

**Table 2.** General Characteristics of the studied sample. Maracaibo city, Venezuela.

	Female		Male		Total	
	n	%	n	%	n	%
**Age Group** **(years)**						
<30	235	34.8	228	41.5	463	37.8
30–49	253	37.4	220	40.0	473	38.6
≥50	188	27.8	102	18.5	290	23.7
**Ethnic Groups**						
Mixed	512	75.7	427	77.6	939	76.6
White Hispanic	111	16.4	80	14.5	191	15.6
Afrodescendant	15	2.2	21	3.8	36	2.9
Indian-American	30	4.4	21	3.8	51	4.2
Other	8	1.2	1	0.2	9	0.7
**Socioeconomic** **Status**						
Class I	15	2.2	9	1.6	24	2.0
Class II	116	17.2	113	20.5	229	18.7
Class III	253	37.4	237	43.1	490	40.0
Class IV	251	37.1	172	31.3	423	34.5
Class V	41	6.1	19	3.5	60	4.9
**Smoking Habit**						
No Smoker	523	77.5	351	64.3	874	71.6
Smoker	76	11.3	105	19.2	181	14.8
Past Smoker	76	11.3	90	16.5	166	13.6
**Hypertension** ‡	126	18.6	144	26.2	270	22.0
**Elevated** **Triglycerides**	139	20.6	170	30.9	309	25.2
**Low HDL-C**	429	63.5	270	49.1	699	57.0
**Metabolic** **Syndrome** *	250	37.0	233	42.4	483	39.4
**Insulin** **Resistance** †	317	46.9	257	46.7	574	46.8
**Total**	**676**	**100.0**	**550**	**100.0**	**1226**	**100.0**

‡ Past history and Diagnosed in the Study * Metabolic Syndrome Diagnosis according to 2009 Harmonizing Consensus † HOMA2-IR ≥2

### Metabolic phenotypes and sociodemographic characteristics

In the evaluation of the epidemiologic behavior of the metabolic phenotypes according to sex, we found that HNW and MUO individuals were predominately females (62.5%, n=336; 51.3%, n=211 respectively), while the atypical phenotypes were predominately males (MUNW: 56.3%, n=36; MHO: 52.6%, n=112. *χ* ^2^=22.53, p<0.001). Likewise, a statistically significant association was found between age groups and metabolic phenotypes ( *χ* ^2^= 211.91, p<0.001), observing a predominance in the < 30 years age group in the normal-weight phenotype (HNW: 56.1%, n=302; MUNW: 57.8%, n=37), whereas the 30–49 age group was predominately obese phenotypes (MHO: 47.9%, n=102; MUO: 50.1%, n=106). There was no statistically significant association between metabolic phenotypes, ethnic groups *(χ* ^2^= 20.96, p=0.05) and socioeconomic status *(χ* ^2^= 14.56, p=0.27) ( Table 3).

**Table 3.** Sociodemographic characteristics according to metabolic phenotypes. Maracaibo city, Venezuela.

	(HNW) A		(MUNW) B		(MHO) C		(MUO) D			A vs. B	A vs. C	A vs. D	B vs. C	B vs. D	C vs. D
	n	%	n	%	n	%	n	%	χ2 ( *p*) *	*p* **	*p* **	*p* **	*p* **	*p* **	*p* **
**Gender**									22.53 (<0.001)						
Female	336	62.5	28	43.8	101	47.4	211	51.3		<0.05	<0.05	<0.05	NS	NS	NS
Male	202	37.5	36	56.3	112	52.6	200	48.7		<0.05	<0.05	<0.05	NS	NS	NS
**Age Group** **(years)**									176.63 (<0.001)						
<30	302	56.1	37	57.8	46	21.6	78	19.0		NS	<0.05	<0.05	<0.05	<0.05	NS
30–49	153	28.4	12	18.8	102	47.9	206	50.1		NS	<0.05	<0.05	<0.05	<0.05	NS
≥50	83	15.5	15	23.4	65	30.5	127	30.9		NS	<0.05	<0.05	NS	NS	NS
**Ethnic Group**									20.96 (0.05)						
Mixed	412	76.6	50	78.1	169	79.3	308	74.9		NS	NS	NS	NS	NS	NS
White Hispanic	74	13.8	6	9.4	31	14.6	80	19.5		NS	NS	NS	NS	NS	NS
Afrodescendant	16	3.0	3	4.7	6	2.8	11	2.7		NS	NS	NS	NS	NS	NS
Indian-American	32	5.9	5	7.8	6	2.8	8	1.9		NS	NS	<0.05	NS	<0.05	NS
Others	4	0.7	0	0.0	1	0.5	4	1.0		NS	NS	NS	NS	NS	NS
**Socioeconomic** **Status**									14.56 (0.27)						
Class I	12	2.2	0	0.0	2	0.9	10	2.4		NS	NS	NS	NS	NS	NS
Class II	96	17.8	15	23.4	35	16.4	83	20.2		NS	NS	NS	NS	NS	NS
Class III	213	39.6	21	32.8	102	47.9	154	37.5		NS	NS	NS	NS	NS	NS
Class IV	187	34.8	25	39.1	62	29.1	149	36.3		NS	NS	NS	NS	NS	NS
Class V	30	5.6	3	4.7	12	5.6	15	3.6		NS	NS	NS	NS	NS	NS
**Total**	**538**	**100**	**64**	**100**	**213**	**100**	**411**	**100**							

HNW (Healthy Normal Weight); MUNW (Metabolically Unhealthy Normal Weight); MHO (Metabolically Healthy Obese); MUO (Metabolically Unhealthy Obese). * Chi-Square Test. ** Z-test of proportions.

### Metabolic phenotypes and psychobiologic habits

Initially, in relation to the smoking habit, the non-smokers were the most frequent group ( *χ ^2^=*30.91; p<0.001), despite the fact MUNW phenotype consisted of the highest percentage of smoking individuals (18.8%, n=12), whereas MUO subjects consisted of the highest proportion of past smoking subjects (20.2%, n=83). On the other side, in the evaluation of the metabolic phenotypes according to PA there was a statistically significant association in the transport-related physical activity ( *χ ^2^=*43.39; p<0.001) and leisure activities ( *χ ^2^=*50.48; p<0.001) ( Table 4).

**Table 4.** Psychobiologic Habits according to metabolic phenotypes. Maracaibo city, Venezuela.

	(HNW) A		(MUNW) B		(MHO) C		(MUO) D			A vs. B	A vs. C	A vs. D	B vs. C	B vs. D	C vs. D
	n	%	n	%	n	%	n	%	χ2 ( *p*) *	*p* **	*p* **	*p* **	*p* **	*p* **	*p* **
**Smoking Habit**									30.91 (<0.001)						
No Smoker	415	77.7	44	68.8	154	72.6	261	63.5		NS	NS	<0.05	NS	NS	NS
Smoker	72	13.5	12	18.8	30	14.2	67	16.3		NS	NS	NS	NS	NS	NS
Past Smoker	47	8.8	8	12.5	28	13.2	83	20.2		NS	NS	<0.05	NS	NS	NS
**Physical Activity** **Work Sphere**									14.17 (0.51)						
Inactive	408	75.8	50	78.1	159	74.6	307	74.7		NS	NS	NS	NS	NS	NS
Very Low	30	5.6	3	4.7	10	4.7	18	4.4		NS	NS	NS	NS	NS	NS
Low	32	5.9	5	7.8	12	5.6	17	4.1		NS	NS	NS	NS	NS	NS
Moderate	25	4.6	2	3.1	7	3.3	21	5.1		NS	NS	NS	NS	NS	NS
High	26	4.8	3	4.7	9	4.2	26	6.3		NS	NS	NS	NS	NS	NS
Very High	17	3.2	1	1.6	16	7.5	22	5.4		NS	NS	NS	NS	NS	NS
**Physical Activity** **Transport Sphere**									43.39 (<0.001)						
Inactive	163	30.6	19	30.2	87	41.0	188	46.4		NS	<0.05	<0.05	NS	NS	NS
Very Low	58	10.9	3	4.8	32	15.1	31	7.7		NS	NS	NS	NS	NS	<0.05
Low	70	13.2	7	11.1	25	11.8	47	11.6		NS	NS	NS	NS	NS	NS
Moderate	73	13.7	9	14.3	26	12.3	44	10.9		NS	NS	NS	NS	NS	NS
High	90	16.9	14	22.2	25	11.8	47	11.6		NS	NS	NS	NS	NS	NS
Very High	78	14.7	11	17.5	17	8.0	48	11.9		NS	NS	NS	NS	NS	NS
**Physical Activity** **Household Sphere**									24.33 (0.06)						
Inactive	125	23.2	15	23.4	75	35.2	126	30.7		NS	<0.05	NS	NS	NS	NS
Very Low	95	17.7	11	17.2	22	10.3	48	11.7		NS	NS	NS	NS	NS	NS
Low	86	16.0	9	14.1	24	11.3	60	14.6		NS	NS	NS	NS	NS	NS
Moderate	83	15.4	11	17.2	38	17.8	63	15.3		NS	NS	NS	NS	NS	NS
High	73	13.6	10	15.6	21	9.9	54	13.1		NS	NS	NS	NS	NS	NS
Very High	76	14.1	8	12.5	33	15.5	60	14.6		NS	NS	NS	NS	NS	NS
**Physical Activity** **Leisure Sphere**									50.48 (<0.001)						
Inactive	305	56.7	37	57.8	134	62.9	290	70.6		NS	NS	NS	NS	NS	NS
Very Low	42	7.8	7	10.9	10	4.7	27	6.6		NS	NS	NS	NS	NS	NS
Low	43	8.0	2	3.1	23	10.8	26	6.3		NS	NS	NS	NS	NS	NS
Moderate	43	8.0	3	4.7	19	8.9	31	7.5		NS	NS	NS	NS	NS	NS
High	41	7.6	6	9.4	20	9.4	21	5.1		NS	NS	NS	NS	NS	NS
Very High	64	11.9	9	14.1	7	3.3	16	3.9		NS	<0.05	<0.05	<0.05	<0.05	NS
**Total**	**538**	**100**	**64**	**100**	**213**	**100**	**411**	**100**							

HNW (Healthy Normal Weight); MUNW (Metabolically Unhealthy Normal Weight); MHO (Metabolically Healthy Obese); MUO (Metabolically Unhealthy Obese). * Chi-Square Test. ** Z-test of proportions.

### Phenotypes and endocrine-metabolic alterations

Distribution of subjects according to phenotypes and endocrine-metabolic alterations are shown in Table 5. A high percentage of MUNW and MUO individuals with insulin resistance was found in contrast to healthy subjects (79.7%, n=51 and 97.1%, n=399, respectively). On the other side, a higher percentage of MUNW with high TAG was found (34.4% n=22 vs 9.5% n=51 HNW; p<0.05) and also a higher prevalence of MS (29.7% n=19 vs 12.3% n=66; p<0.05 HNW); similar findings were observed in the obese phenotypes, where a minor prevalence of these alterations were found in the MHO subjects (high TAG levels: 28.8% n=60 vs 42.8% n=176, p<0.05; MS: 53.1% n=113 vs 69.3% n=285, p<0.05). Finally, a significant association was found between the metabolic phenotypes with low HDL-C ( *χ* ^2^=44.08; p<0.0001) and HTN ( *χ* ^2^= 182.22, p<0.0001).

**Table 5.** Endocrine-Metabolic Alterations according to metabolic phenotypes Maracaibo city, Venezuela.

	(HNW) A		(MUNW) B		(MHO) C		(MUO) D			A vs. B	A vs. C	A vs. D	B vs. C	B vs. D	C vs. D
	n	%	n	%	n	%	n	%	χ2 ( *p*) *	*p* **	*p* **	*p* **	*p* **	*p* **	*p* **
**HOMA2-IR**									727.9 (<0.0001)						
<2	434	80.7	13	20.3	193	90.6	12	2.9		<0.05	NS	<0.05	NS	<0.05	<0.05
≥2	104	19.3	51	79.7	20	9.4	399	97.1		<0.05	<0.05	<0.05	<0.05	<0.05	<0.05
**Hypertension**									182.22 (<0.0001)						
Absent	331	87.3	32	82.1	53	43.1	96	39.8		NS	<0.05	<0.05	<0.05	<0.05	NS
Present ‡	48	12.7	7	17.9	70	56.9	145	60.2		NS	<0.05	<0.05	<0.05	<0.05	NS
**Triglycerides**									142.09 (<0.0001)						
Normal	487	90.5	42	65.6	153	71.8	235	57.2		<0.05	<0.05	<0.05	NS	NS	<0.05
High	51	9.5	22	34.4	60	28.2	176	42.8		<0.05	<0.05	<0.05	NS	NS	<0.05
**HDL-C**									44.08 (<0.0001)						
Normal	283	52.6	30	46.9	85	39.9	129	31.4		NS	<0.05	<0.05	NS	NS	NS
Low	255	47.4	34	53.1	128	60.1	282	68.6		NS	<0.05	<0.05	NS	NS	NS
**Metabolic** **Syndrome**									339.38 (<0.0001)						
Absent	472	87.7	45	70.3	100	46.9	126	30.7		<0.05	<0.05	<0.05	<0.05	<0.05	<0.05
Present	66	12.3	19	29.7	113	53.1	285	69.3		<0.05	<0.05	<0.05	<0.05	<0.05	<0.05
**Total**	**538**	**100**	**64**	**100**	**213**	**100**	**411**	**100**							

HNW (Healthy Normal Weight); MUNW (Metabolically Unhealthy Normal Weight); MHO (Metabolically Healthy Obese); MUO (Metabolically Unhealthy Obese).

* Chi-Square Test.

** Z-test of proportions.

‡Personal history and Diagnosis in the Study

### Metabolic phenotypes and biologic-anthropometric variables

Biochemical and clinical characteristics according to metabolic phenotypes are shown in Table 6. An increasing tendency of their variable levels was observed, except on HOMA2-IR, HOMA2-βcell, HOMA2-S, insulin y glucose levels.

**Table 6.** Clinical and biochemical characteristics according to metabolic phenotypes. Maracaibo city, Venezuela.

	HNW		MUNW		MHO		MUO		
	Mean	SD	Mean	SD	Mean	SD	Mean	SD	*p* *
**Age (years)**	32,5	14,7	34,1	16,5	42,9	13,5	43,1	13,2	<0.001
**Body Mass Index** **(Kg/m ^2^)**	21,9	2,1	22,9	1,7	34,5	4,7	35,4	5,6	<0.001
**Waist Circunference (cm)**									
Female	79,3	8,2	77,2	7,1	104,4	10,6	105,5	10,1	<0.001
Male	81,5	6,9	86,9	7,6	109,2	11,9	116,0	15,3	<0.001
**HOMA2-βcell**	127,2	40,4	204,5	88,2	118,9	37,0	188,7	80,8	<0.001
**HOMA2-S**	81,9	44,6	41,0	27,3	80,6	36,9	32,8	10,5	<0.001
**HOMA2-IR**	1,5	0,5	3,2	1,6	1,4	0,4	3,5	1,6	<0.001
**Insulin (µU/mL)**	9,9	3,6	22,3	11,9	9,6	2,9	23,7	11,8	<0.001
**Glucose (mg/dL)**	89,3	10,1	94,9	22,7	91,9	11,3	103,2	28,9	<0.001
**Total Cholesterol** **(mg/dL)**	174,9	38,8	180,1	44,9	196,5	52,3	200,8	45,4	<0.001
**Triglycerides (mg/dL)** ¶	73.4	53.0–106.0	99.1	67.9–209.0	107.7	75.0–164.0	135.2	97.0–193.0	<0.001
**HDL-C (mg/dL)**									
Female	49,3	11,8	51,6	11,5	45,6	13,0	44,1	11,5	<0.001
Male	46,0	11,2	39,5	11,8	40,2	9,9	36,7	8,5	<0.001
**VLDL-C (mg/dL)**	17,1	9,3	31,0	28,5	26,7	20,4	32,5	21,5	<0.001
**LDL-C (mg/dL)**	109,8	34,5	106,4	40,2	126,3	35,1	128,0	37,2	<0.001
**Lipoprotein(a) (mg/dL)**	26,1	14,0	22,2	14,7	28,7	13,4	29,3	14,1	<0.001
**hs-C Reactive Protein** **(mg/L)** ¶	0.297	0.070–0.598	0.235	0.099–0.580	0.435	0.177–0.814	0.562	0.195–1.222	<0.001
**Non HDL Cholesterol**	126,9	38,6	135,3	45,5	153,8	51,9	160,3	45,1	<0.001
**Triacylglicerides/** **HDL-C Index** ¶	1.5	1.0–2.4	2.4	1.4–5.5	2.8	1.7–4.1	3.5	2.3–5.5	<0.001
**Visceral Adiposity** **Index** ¶	1.7	0.7–1.8	1.6	0.9–3.3	1.8	1.2–2.9	2.4	1.7–3.9	<0.001
**Systolic Blood** **Pressure (mmHg)**	111,9	13,3	115,2	15,3	125,3	18,4	125,6	17,3	<0.001
**Diastolic Blood** **Pressure (mmHg)**	71,7	9,4	73,9	10,9	81,5	12,3	81,9	11,2	<0.001

HNW (Healthy Normal Weight); MUNW (Metabolically Unhealthy Normal Weight); MHO (Metabolically Healthy Obese); MUO (Metabolically Unhealthy Obese).

SD=Standar Deviation;

* One-way ANOVA Test

¶ As Median (p25–p75 ^th^) Comparison: Kruskal Wallis Test

### Metabolic phenotypes and coronary risk classification

An association between metabolically unhealthy phenotypes and a higher risk of a coronary event was found. This association was stronger for unhealthy phenotypes than for their healthy counterparts. However, results were statistically significant for obese individuals (MHO: OR=1.85 CI95%: 1.11-3.09; p=0.02 and MUO: OR=2.09 CI95%: 1.34-3.28; p<0.01) ( Table 7).

**Table 7.** Logistic regression model for metabolic phenotypes and coronary risk categories. Maracaibo city, Venezuela.

	Crude Odds Ratio (IC 95% ^a^)	*p* ^b^	Adjusted Odds Ratio * (IC 95% ^a^)	*p* ^b^
**Metabolic Phenotypes**				
Metabolically Healthy Normal Weight	1,00	-	1,00	-
Metabolically Unhealthy Normal Weight	3,41 (1,46 - 7,98)	< 0,01	2.24 (0,89 - 5.56)	0,08
Metabolically Healthy Obese	2,26 (1,40 - 3,64)	< 0,01	1.85 (1.11 - 3.09)	0,02
Metabolically Unhealthy Obese	2,85 (1,89 - 4,29)	< 0,01	2.09 (1.34 - 3.28)	< 0,01

**a** Confidence Interval (95%); **b** Level of significance Dependent Variable: Coronary risk: <5% in 10 years vs ≥5% in 10 years * Adjusted Model for: sex, age, ethnic group, socioeconomic status, smoking habit, physical activity in leisure dimension according to IPAQ, high TAG, and metabolic phenotypes.

MMSPS metabolic phenotype dataset BMI: Body Mass Index, WaistC: Waist Circumference, HDL-C: High Density Lipoprotein Cholesterol, VLDL-C: Very Low Density Lipoprotein Cholesterol, LDL-C: Low Density Lipoprotein Cholesterol, Lp(a): Lipoprotein (a), hs-CRP: high Sensitivity C Reactive Protein, Non-HDL-Col: Non-High Density Lipoprotein Cholesterol, TAG/HDL ratio: Triglycerides/High Density Lipoprotein ratio VAI: Visceral Adiposity Index, BP: Blood Pressure, HNW: Healthy Normal-Weight, MUNW: Metabolically Unhealthy Normal-Weight, MUO: Metabolically Unhealthy Obese, MHO: Metabolically Healthy Obese. Click here for additional data file. Copyright: © 2018 Bermudez V et al. 2018 Data associated with the article are available under the terms of the Creative Commons Zero "No rights reserved" data waiver (CC0 1.0 Public domain dedication).

## Discussion

Obesity is a prioritized area for the world health systems because of its increasing prevalence, incidence, and associated costs in the last decade ^20^. This disease has been defined classically as “excessive presence of adipose tissue that is injurious for health” and given its association to other chronic-degenerative diseases ^3, 21^ has been stereotyped as “more adiposity, more risk”. All the classic methods employed for obesity diagnosis, even central and global, are indirect measurements. For different populations they do not allow to determine the adipose tissue functioning from individuals, even though they have high sensitivity, specificity, and predictive values. Based on this, multiple epidemiologic studies have detected a considerable percentage of individuals who did not enter in the classic “HNW” and “MUO” phenotypes, showing the existence of atypical metabolic phenotypes called “MUNW” and “MHO” ^3^. The defining criteria of these metabolic states differ significantly between studies and are defined under highly subjectivity levels, nonetheless insulin sensitivity and lipid profile are often used to define healthy and unhealthy phenotypes ^22– 24^.

Giving this criteria and methods discrepancy, such as the psychobiologic, sociodemographic, and genetic patterns according to latitudes, the phenotype frequency presents high variability ^25^. This could bias the study by selecting predetermined variables and cut-off points to consider an individual as healthy or unhealthy. In this sense, data mining techniques were proposed to avoid potential bias. The program would group subjects according to spontaneous tendencies and biologic behavior of related variables.

Applied studies in Asia reported a prevalence of 8.7%–13.07% and 3.9%–15.5% for MUNW and MHO phenotypes, respectively ^26, 27^. Likewise, studies conducted in Europe reported frequencies ranging between 18.9% and 45.8% for the MUNW phenotype, and between 2.1% and 18.5% for the MHO phenotype ^28– 30^; a similar variability was observed in American research studies ^31, 32^. Latin American reports are scant, however Fanghanel *et al.* ^33^ showed a 5.8% prevalence of the MUNW phenotype for the Mexico City, similar to the one showed in the present study, whereas contrasting the obese phenotypes the Maracaibo population exhibited the highest prevalence of MHO subjects (17% vs 10.8% of the Mexican population).

The atypical metabolic phenotypes, as MUNW and MHO, tend to be observed in females with more frequency ^32, 34^. However, the present study reported these phenotypes were more frequent in males. Significant difference between sexes was found in the MUNW group, similar to the study by Hinnouko *et al*. ^35^. Smoking habit, age, and physical activity values, were discovered as influencing factors in these findings.

In the same manner, multiple studies have reported that healthy phenotype prevalence decreases with age ^27, 29^, but in our population an increase was observed in the frequency of MHO individuals older than 30 years old. Yoo *et al.* ^36^ did not report differences in this phenotype prevalence between subjects older and younger than 35 years. Regarding the MUNW phenotype in the Maracaibo population, a higher frequency was found in subjects younger than 30 years. A considerable part of epidemiologic studies that evaluate this association possessed samples conformed by subjects older than 35 years. This may limit the establishment of a tendency in frequency of healthy phenotypes according to age. Similarly, factors such as ethnicity from African descendants ^37^ and socioeconomical status ^38^ have been related to the presence of atypical phenotypes, but no relationship was found between these variables in Maracaibo population.

One of the greatest enigmas formulated in relation to the atypical metabolic phenotypes, is focused on its conditioning factors. Psychobiologic habits have been considered key elements in comprehension of its biology and behavior related to time. Diniz *et al.* ^39^ found a significant association between healthy metabolic phenotypes with absence of smoking habit, also with increased PA levels, such as the present study. Ortega *et al.* ^40^ reported that MHO subjects present with better cardiorespiratory fitness profiles than their unhealthy counterpart, and by adjusting for this variable the MHO individuals showed less mortality. Other studies report that the phenotypes progression from health to unhealthiness is not related to the smoking habit, alcohol, or quantified PA through indirect methods ^30^ and depends fundamentally on abdominal circumference and visceral adiposity increment.

Regarding to cardiometabolic profiles, our study showed evidence of significantly higher HOMA2-βcell values in all of the unhealthy phenotypes, described previously by the NHANES study ^41^ and by Madeira *et al.* ^42^. Also higher HOMA2-IR and a lower HOMA2-S demonstrate again the importance to define metabolic states in lean and obese individuals. They could also elevate the risk of developing T2DM and CVD in the unhealthy phenotypes, given their hyper functioning pancreatic beta cell and hyperinsulinemia ^43^.

MHO subjects present with lower HOMA2-IR and higher TAG, LDL-C, PAS, PAD, and hs-CRP levels. In contrast to lean subjects, MHO has higher VAI. The latter constitutes an initial obesity state, without a significant risk of T2DM and CVD in the short term (7–11 years) ^44^, but there is in the long term (>16–30 years) ^45^. The natural history of the MHO is variable, only 16% of MHO individuals stay on that status without alteration for the following 7–8 years ^46^. Those who progress to an unhealthy state present a higher risk of high blood pressure, low-grade inflammation, bad metabolic control and high TAG ^30^. In spite of the metabolic “benign” state of the MHO adipose tissue, non-metabolic complications of obesity, do not exclude these subjects from getting T2DM, CVD, and chronic diseases associated with obesity in the future ^34, 35^.

Healthy obese individuals must be classified in categories with higher risk of a coronary event compared to lean subjects. This is consistent with previous reports related to metabolic phenotypes and CVD, suggesting that healthy obese subjects have a higher risk profile in comparison to those with lower BMI ^36^; as well as an increased risk for CVD ^47^ and metabolic disorders such as fatty liver and low-grade inflammation ^48^. Given the above, a profound evaluation of these patients is recommended. This includes not only obese subjects but also those who are overweight, which can go unnoticed in a routine consultation and CVD could be subclinical; as it has been demonstrated by Khan *et al.* in 475 women from the SWAN study ^49^.

Finally, despite the fact that our report presents a novel method to classify healthy and unhealthy subjects, it is important to mention the difficulty to follow-up these individuals. The latter would show the atypical phenotype stability related to time, as well as the incidence of T2DM and CVD. This was the main limitation of our study. In addition our study lacks nutritional data. For this reason, a thorough and constant evaluation of subjects with atypical metabolic phenotypes is recommended, given their demonstrated unsteadiness in time, and associated non metabolic comorbidities observed especially in the MHO individuals.

## Data availability

The data referenced by this article are under copyright with the following copyright statement: Copyright: ï¿½ 2018 Bermudez V et al.

Data associated with the article are available under the terms of the Creative Commons Zero "No rights reserved" data waiver (CC0 1.0 Public domain dedication). 

Dataset 1: **MMSPS metabolic phenotype dataset.** BMI: Body Mass Index, WaistC: Waist Circumference, HDL-C: High Density Lipoprotein Cholesterol, VLDL-C: Very Low Density Lipoprotein Cholesterol, LDL-C: Low Density Lipoprotein Cholesterol, Lp(a): Lipoprotein (a), hs-CRP: high Sensitivity C Reactive Protein, Non-HDL-Col: Non-High Density Lipoprotein Cholesterol, TAG/HDL ratio: Triglycerides/High Density Lipoprotein ratio VAI: Visceral Adiposity Index, BP: Blood Pressure, HNW: Healthy Normal-Weight, MUNW: Metabolically Unhealthy Normal-Weight, MUO: Metabolically Unhealthy Obese, MHO: Metabolically Healthy Obese. 10.5256/f1000research.13897.d193351 ^50^

## Ethics and consent

The study was approved by the Bioethics Committee of the Endocrine and Metabolic Research Center – University of Zulia (approval number: BEC-006-0305). This ethical approval included all future studies that used the data from the Maracaibo City Metabolic Syndrome Prevalence Study (MMSPS). All participants signed written a informed consent for participation in the study before being questioned and physically examined by a trained team.

## Abbreviations

**CVD: cardiovascular disease**

**HDL-C: High Density Lipoprotein - Cholesterol**

**HNW: healthy normal-weight**

**HOMA: Homeostasis Model Assesment**

**HTN: hypertension**

**hs-CRP: high-sensitivity C-Reactive Protein**

**IR: insulin resistance**

**LDL-C: Low Density Lipoprotein – Cholesterol**

**MAP: mean arterial pressure**

**MET: Metabolic Equivalent**

**MMSPS: Maracaibo City Metabolic Syndrome Prevalence Study**

**MHO: metabolically healthy obese**

**MS: Metabolic Syndrome**

**MUNW: metabolically unhealthy normal-weight**

**MUO: metabolically unhealthy obese**

**PA: Physical activity**

**SD: standard deviation**

**TAG: triglycerides**

**T2DM: Type 2 Diabetes Mellitus**

**VAI: Visceral Adiposity Index**

## References

[1] World Health Organization (WHO): Obesity and overweight: Fact Sheet.2016; [cited 2017 Mar 20]. Reference Source

[2] Center for Disease Control and Prevention (CDC): Adult Obesity Causes & Consequences | Overweight & Obesity.2016; [cited 2017 Mar 20]. Reference Source

[3] Andres R : Effect of obesity on total mortality. *Int J Obes.* 1980;4(4):381–6. 6998887

[4] Ferrannini E Natali A Bell P : Insulin resistance and hypersecretion in obesity. European Group for the Study of Insulin Resistance (EGIR). *J Clin Invest.* 1997;100(5):1166–73. 10.1172/JCI119628 9303923 PMC508292

[5] Bernstein RS Grant N Kipnis DM : Hyperinsulinemia and enlarged adipocytes in patients with endogenous hyperlipoproteinemia without obesity or diabetes mellitus. *Diabetes.* 1975;24(2):207–13. 10.2337/diab.24.2.207 1123108

[6] Ruderman NB Schneider SH Berchtold P : The “metabolically-obese,” normal-weight individual. *Am J Clin Nutr.* 1981;34(8):1617–21. 10.1093/ajcn/34.8.1617 7270486

[7] Shaharyar S Roberson LL Jamal O : Obesity and metabolic phenotypes (metabolically healthy and unhealthy variants) are significantly associated with prevalence of elevated C-reactive protein and hepatic steatosis in a large healthy Brazilian population. *J Obes.* 2015;2015: 178526. 10.1155/2015/178526 25838943 PMC4369939

[8] Bermúdez V Marcano RP Cano C : The Maracaibo city metabolic syndrome prevalence study: design and scope. *Am J Ther.* 2010;17(3):288–94. 10.1097/MJT.0b013e3181c121bc 20068446

[9] Bermúdez V Rojas J Salazar J : Sensitivity and Specificity Improvement in Abdominal Obesity Diagnosis Using Cluster Analysis during Waist Circumference Cut-Off Point Selection. *J Diabetes Res.* 2015;2015: 750265. 10.1155/2015/750265 25945356 PMC4402167

[10] Mendez Castellano H de Mendez MC : Estratificacion social y biologia humana: metodo Graffar modificado. *Arch Venez Pueric Pediatría.* 1986;49(3–4):93–104. Reference Source

[11] Chobanian AV Bakris GL Black HR : The Seventh Report of the Joint National Committee on Prevention, Detection, Evaluation, and Treatment of High Blood Pressure: the JNC 7 report. *JAMA.* 2003;289(19):2560–72. 10.1001/jama.289.19.2560 12748199

[12] World Health Organization (WHO): The world health report 2003 - shaping the future.2003; [cited 2017 Nov 20]. Reference Source

[13] Center for Disease Control and Prevention (CDC): Health Statistics, NHANES III Reference Manuals and Reports.1996; [cited 2017 Nov 20]. Reference Source

[14] Bermudez V Salazar J Martínez MS : Prevalence and Associated Factors of Insulin Resistance in Adults from Maracaibo City, Venezuela. *Adv Prev Med.* 2016;2016: 9405105. 10.1155/2016/9405105 27579182 PMC4989131

[15] Friedewald WT Levy RI Fredrickson DS : Estimation of the concentration of low-density lipoprotein cholesterol in plasma, without use of the preparative ultracentrifuge. *Clin Chem.* 1972;18(6):499–502. 4337382

[16] Levy JC Matthews DR Hermans MP : Correct homeostasis model assessment (HOMA) evaluation uses the computer program. *Diabetes Care.* 1998;21(12):2191–2. 10.2337/diacare.21.12.2191 9839117

[17] Amato MC Giordano C Galia M : Visceral Adiposity Index: a reliable indicator of visceral fat function associated with cardiometabolic risk. *Diabetes Care.* 2010;33(4):920–2. 10.2337/dc09-1825 20067971 PMC2845052

[18] Alberti KG Eckel RH Grundy SM : Harmonizing the metabolic syndrome: a joint interim statement of the International Diabetes Federation Task Force on Epidemiology and Prevention; National Heart, Lung, and Blood Institute; American Heart Association; World Heart Federation; International Atherosclerosis Society; and International Association for the Study of Obesity. *Circulation.* 2009;120(16):1640–5. 10.1161/CIRCULATIONAHA.109.192644 19805654

[19] Bermúdez V Salazar J Bello L : Coronary Risk Estimation According to a Recalibrated Framingham-Wilson Score in the Maracaibo City Metabolic Syndrome Prevalence Study. *J Cardiol Photon.* 2014;107:160–8. Reference Source

[20] World Health Organization (WHO): Global action plan for the prevention and control of NCDs 2013–2020.2013; [cited 2017 Nov 20]. Reference Source

[21] Hubert HB Feinleib M McNamara PM : Obesity as an independent risk factor for cardiovascular disease: a 26-year follow-up of participants in the Framingham Heart Study. *Circulation.* 1983;67(5):968–77. 10.1161/01.CIR.67.5.968 6219830

[22] Brochu M Tchernof A Dionne IJ : What are the physical characteristics associated with a normal metabolic profile despite a high level of obesity in postmenopausal women? *J Clin Endocrinol Metab.* 2001;86(3):1020–5. 10.1210/jcem.86.3.7365 11238480

[23] Stefan N Kantartzis K Machann J : Identification and characterization of metabolically benign obesity in humans. *Arch Intern Med.* 2008;168(15):1609–16. 10.1001/archinte.168.15.1609 18695074

[24] Aguilar-Salinas CA García EG Robles L : High adiponectin concentrations are associated with the metabolically healthy obese phenotype. *J Clin Endocrinol Metab.* 2008;93(10):4075–9. 10.1210/jc.2007-2724 18682512

[25] Martínez-Larrad MT Corbatón Anchuelo A Del Prado N : Profile of individuals who are metabolically healthy obese using different definition criteria. A population-based analysis in the spanish population. *PLoS One.* 2014;9(9):e106641. 10.1371/journal.pone.0106641 25198070 PMC4157807

[26] Lee K : Metabolically obese but normal weight (MONW) and metabolically healthy but obese (MHO) phenotypes in Koreans: characteristics and health behaviors. *Asia Pac J Clin Nutr.* 2009;18(2):280–4. 19713189

[27] Zheng R Yang M Bao Y : Prevalence and Determinants of Metabolic Health in Subjects with Obesity in Chinese Population. *Int J Environ Res Public Health.* 2015;12(11):13662–77. 10.3390/ijerph121113662 26516886 PMC4661606

[28] Calori G Lattuada G Piemonti L : Prevalence, metabolic features, and prognosis of metabolically healthy obese Italian individuals: the Cremona Study. *Diabetes Care.* 2011;34(1):210–5. 10.2337/dc10-0665 20937689 PMC3005463

[29] Lopez-Garcia E Guallar-Castillon P Leon-Muñoz L : Prevalence and determinants of metabolically healthy obesity in Spain. *Atherosclerosis.* 2013;231(1):152–7. 10.1016/j.atherosclerosis.2013.09.003 24125427

[30] Hamer M Bell JA Sabia S : Stability of metabolically healthy obesity over 8 years: the English Longitudinal Study of Ageing. *Eur J Endocrinol.* 2015;173(5):703–8. 10.1530/EJE-15-0449 26286585

[31] Kuk JL Ardern CI : Are metabolically normal but obese individuals at lower risk for all-cause mortality? *Diabetes Care.* 2009;32(12):2297–9. 10.2337/dc09-0574 19729521 PMC2782994

[32] Wildman RP Muntner P Reynolds K : The obese without cardiometabolic risk factor clustering and the normal weight with cardiometabolic risk factor clustering: prevalence and correlates of 2 phenotypes among the US population (NHANES 1999–2004). *Arch Intern Med.* 2008;168(15):1617–24. 10.1001/archinte.168.15.1617 18695075

[33] Fanghänel-Salmón G Gutiérrez-Salmeán G Samaniego V : Obesity Phenotypes In Urban Middle-Class Cohorts; The Prit-Lindavista Merging Evidence In Mexico: The Opus Prime Study. *Nutr Hosp.* 2015;32(1):182–8. 10.3305/nh.2015.32.1.8646 26262714

[34] Bo S Musso G Gambino R : Prognostic implications for insulin-sensitive and insulin-resistant normal-weight and obese individuals from a population-based cohort. *Am J Clin Nutr.* 2012;96(5):962–9. 10.3945/ajcn.112.040006 23034958

[35] Hinnouho GM Czernichow S Dugravot A : Metabolically healthy obesity and the risk of cardiovascular disease and type 2 diabetes: the Whitehall II cohort study. *Eur Heart J.* 2015;36(9):551–9. 10.1093/eurheartj/ehu123 24670711 PMC4344958

[36] Yoo HK Choi EY Park EW : Comparison of Metabolic Characteristics of Metabolically Healthy but Obese (MHO) Middle-Aged Men According to Different Criteria. *Korean J Fam Med.* 2013;34(1):19–26. 10.4082/kjfm.2013.34.1.19 23372902 PMC3560335

[37] Schmiegelow MD Hedlin H Mackey RH : Race and ethnicity, obesity, metabolic health, and risk of cardiovascular disease in postmenopausal women. *J Am Heart Assoc.* 2015;4(5): pii:e001695. 10.1161/JAHA.114.001695 25994446 PMC4599406

[38] Yang HK Han K Kwon HS : Obesity, metabolic health, and mortality in adults: a nationwide population-based study in Korea. *Sci Rep.* 2016;6(6): 30329. 10.1038/srep30329 27445194 PMC4957204

[39] Diniz Mde F Beleigoli AM Ribeiro AL : Factors associated with metabolically healthy status in obesity, overweight, and normal weight at baseline of ELSA-Brasil. *Medicine (Baltimore).* 2016;95(27):e4010. 10.1097/MD.0000000000004010 27399079 PMC5058808

[40] Ortega FB Lee DC Katzmarzyk PT : The intriguing metabolically healthy but obese phenotype: cardiovascular prognosis and role of fitness. *Eur Heart J.* 2013;34(5):389–97. 10.1093/eurheartj/ehs174 22947612 PMC3561613

[41] Romero-Corral A Somers VK Sierra-Johnson J : Normal weight obesity: a risk factor for cardiometabolic dysregulation and cardiovascular mortality. *Eur Heart J.* 2010;31(6):737–46. 10.1093/eurheartj/ehp487 19933515 PMC2838679

[42] Madeira FB Silva AA Veloso HF : Normal weight obesity is associated with metabolic syndrome and insulin resistance in young adults from a middle-income country. *PLoS One.* 2013;8(3):e60673. 10.1371/journal.pone.0060673 23556000 PMC3610876

[43] Succurro E Marini MA Frontoni S : Insulin secretion in metabolically obese, but normal weight, and in metabolically healthy but obese individuals. *Obes (Silver Spring).* 2008;16(8):1881–6. 10.1038/oby.2008.308 18551117

[44] Ogorodnikova AD Kim M McGinn AP : Incident cardiovascular disease events in metabolically benign obese individuals. *Obes (Silver Spring).* 2012;20(3):651–9. 10.1038/oby.2011.243 21799477 PMC3494999

[45] Hansen L Netterstrøm MK Johansen NB : Metabolically Healthy Obesity and Ischemic Heart Disease: A 10-Year Follow-Up of the Inter99 Study. *J Clin Endocrinol Metab.* 2017;102(6):1934–1942. 10.1210/jc.2016-3346 28323999

[46] Kim NH Seo JA Cho H : Risk of the Development of Diabetes and Cardiovascular Disease in Metabolically Healthy Obese People: The Korean Genome and Epidemiology Study. *Medicine (Baltimore).* 2016;95(15):e3384. 10.1097/MD.0000000000003384 27082607 PMC4839851

[47] Durward CM Hartman TJ Nickols-Richardson SM : All-cause mortality risk of metabolically healthy obese individuals in NHANES III. *J Obes.* 2012;2012: 460321. 10.1155/2012/460321 23304462 PMC3523154

[48] Shaharyar S Roberson LL Jamal O : Obesity and metabolic phenotypes (metabolically healthy and unhealthy variants) are significantly associated with prevalence of elevated C-reactive protein and hepatic steatosis in a large healthy Brazilian population. *J Obes.* 2015;2015: 178526. 10.1155/2015/178526 25838943 PMC4369939

[49] Khan UI Wang D Thurston RC : Burden of subclinical cardiovascular disease in “metabolically benign” and “at-risk” overweight and obese women: the Study of Women’s Health Across the Nation (SWAN). *Atherosclerosis.* 2011;217(1):179–86. 10.1016/j.atherosclerosis.2011.01.007 21310415 PMC3117052

[50] Bermudez V Rojas J Salazar J : Dataset 1 in: Biochemical and clinical characterization of metabolic phenotypes: a cross-sectional study from Maracaibo city, Venezuela. *F1000Research.* 2018. 10.5256/f1000research.13897.d193351 PMC8796010 35136588

