# Supplementary material for: Biochemical and clinical characterization of metabolic phenotypes: a cross-sectional study from Maracaibo city, Venezuela
Source: F1000Res. 2021 Mar 24;7:230. Originally published 2018 Feb 27. [Version 3] doi: 10.12688/f1000research.13897.3 (PMC8796010; doi:10.12688/f1000research.13897.3)
Supplement: Peer review history

# Peer review: Reviewer response for version 3

**DOI:** 10.5256/f1000research.29764.r119708

**Published:** 2022-01-27

**Authors:** Hannah XY Hui

**Affiliations:** 1 School of Biomedical Sciences, The Chinese University of Hong Kong, Hong Kong, China

The authors carried out a cross-sectional sub-analysis on The Maracaibo City Metabolic Syndrome Prevalence Study, to evaluate biochemical and clinical characteristics of metabolic phenotypes in Maracaibo city. The prevalence of healthy normal weight, unhealthy normal weight, healthy obese and unhealthy obese subjects was obtained. “Metabolic Unhealthy Normal-Weight” and “Metabolic Healthy Obese” are interesting but has been controversial concepts, which makes the current study potentially important.

 It is interesting to note that the tendency of atypical metabolic phenotypes in either male or female is different in the current cohort than the previously report ones. Could the authors elaborate more in the discussion section in terms of the possible reason and the clinical implications lying behind?

Is the work clearly and accurately presented and does it cite the current literature?

Yes

If applicable, is the statistical analysis and its interpretation appropriate?

I cannot comment. A qualified statistician is required.

Are all the source data underlying the results available to ensure full reproducibility?

Yes

Is the study design appropriate and is the work technically sound?

Yes

Are the conclusions drawn adequately supported by the results?

Yes

Are sufficient details of methods and analysis provided to allow replication by others?

Yes

Reviewer Expertise:

Endocrine disorders, Obesity, Insulin resistance

I confirm that I have read this submission and believe that I have an appropriate level of expertise to confirm that it is of an acceptable scientific standard.

# Peer review: Reviewer response for version 3

**DOI:** 10.5256/f1000research.29764.r82112

**Published:** 2021-03-31

**Authors:** Manfred J. Müller

**Affiliations:** 1 Institute of human nutrition and food science, Christian Albrechts Universität zu Kiel, Kiel, Germany

I still feel that the authors did not specifically address my concerns. To refer to other authors (and their papers) who went the same way of thinking cannot provide an opportunity to do a next step forward. The arbitrary nature of statistically defined phenotypes should be mentioned in the discussion of the paper. It is obvious that we need a functional approach rather than a static and statistical approach to address metabolic phenotypes. Metabolism is a process which cannot be characterized by e.g. assessing the plasma levels of metabolites and hormones in a basal state. By contrast, metabolism is about fluxes and substrate  turnovers which again are related to systemic outcomes (e.g., body temperature, heart rate, blood pressure etc). The concept of functional body composition‘ provides a useful framework of future research. This has to be addressed in the discussion section. Resisting on a conventional approach is not solution-oriented research.

 Repeating and repeating again that many scientists still use crude anthropometric variables cannot be taken as a justification to go on using them. Faced with the present methodological advances it is questionable to go on to apply outdated methods (i.e., anthropometry). Modern techniques used for body composition analysis are neither cumbersome nor expensive. They are already established in huge population studies like NHANES and the UK Biobank Study. If clinicians still insist to use the BMI and wc they are after now. This is a matter of fact. Again, referring to other authors who still work on BMI and wc cannot be taken as a justification to be after.

Is the work clearly and accurately presented and does it cite the current literature?

Yes

If applicable, is the statistical analysis and its interpretation appropriate?

Partly

Are all the source data underlying the results available to ensure full reproducibility?

Yes

Is the study design appropriate and is the work technically sound?

Partly

Are the conclusions drawn adequately supported by the results?

Partly

Are sufficient details of methods and analysis provided to allow replication by others?

Yes

Reviewer Expertise:

Metabolism, phenotyping

I confirm that I have read this submission and believe that I have an appropriate level of expertise to confirm that it is of an acceptable scientific standard, however I have significant reservations, as outlined above.

# Peer review: Reviewer response for version 2

**DOI:** 10.5256/f1000research.19357.r70666

**Published:** 2020-09-03

**Authors:** Manfred J. Müller

**Affiliations:** 1 Institute of human nutrition and food science, Christian Albrechts Universität zu Kiel, Kiel, Germany

My major concern is about the general idea of a metabolically healthy obese subjects. This is an arbitrary rather than a biological phenotype. BMI (and also fat mass) have a limited precision to estimate metabolic risks, i.e., the respective ROC estimates are around 0.7. Since obesity is about categorization (based on observational data on the association between BMI and mortality risk) the finding that about 30% of obese subjects have no measurable metabolic risks question that clinical categorization rather than generating a specific metabolic phenotype worthwhile to study in detail. Thus, the true message of the present paper should be about a metabolically based categorization of the BMI. To get the idea the authors are referred to e.g., Obes Sci & Pract 2017 ^1^.

Is the work clearly and accurately presented and does it cite the current literature?

Yes

If applicable, is the statistical analysis and its interpretation appropriate?

Partly

Are all the source data underlying the results available to ensure full reproducibility?

Yes

Is the study design appropriate and is the work technically sound?

Partly

Are the conclusions drawn adequately supported by the results?

Partly

Are sufficient details of methods and analysis provided to allow replication by others?

Yes

Reviewer Expertise:

Metabolism, phenotyping

I confirm that I have read this submission and believe that I have an appropriate level of expertise to state that I do not consider it to be of an acceptable scientific standard, for reasons outlined above.

## References

[1] : Definition of new cut-offs of BMI and waist circumference based on body composition and insulin resistance: differences between children, adolescents and adults. *Obes Sci Pract* .3(3) : 10.1002/osp4.121 272-281 10.1002/osp4.121 29071103PMC5598017

# Peer review: Reviewer response for version 2

**DOI:** 10.5256/f1000research.19357.r42581

**Published:** 2019-01-22

**Authors:** Víctor A. Cortes

**Affiliations:** 1 Department of Nutrition, Diabetes and Metabolism, School of Medicine, Pontifical Catholic University of Chile (UC), Santiago, Chile

I believe the current version of the paper fully addresses my comments.

Is the work clearly and accurately presented and does it cite the current literature?

Partly

If applicable, is the statistical analysis and its interpretation appropriate?

I cannot comment. A qualified statistician is required.

Are all the source data underlying the results available to ensure full reproducibility?

Yes

Is the study design appropriate and is the work technically sound?

Yes

Are the conclusions drawn adequately supported by the results?

Yes

Are sufficient details of methods and analysis provided to allow replication by others?

Partly

Reviewer Expertise:

NA

I confirm that I have read this submission and believe that I have an appropriate level of expertise to confirm that it is of an acceptable scientific standard.

# Peer review: Reviewer response for version 1

**DOI:** 10.5256/f1000research.15107.r35881

**Published:** 2018-07-19

**Authors:** Víctor A. Cortes

**Affiliations:** 1 Department of Nutrition, Diabetes and Metabolism, School of Medicine, Pontifical Catholic University of Chile (UC), Santiago, Chile

The authors aimed to estimate the prevalence of “atypical metabolic phenotypes” i.e. lean metabolically unhealthy and obese metabolically healthy individuals in the city area of Maracaibo, Venezuela. For that, they analyzed previously generated data from a cross-sectional city-wide health survey (MMSPS), grouping individuals in three BMI categories (normal weight, overweight and obese) and in two “metabolic health” categories (healthy and unhealthy).

 Overall, this study is incremental, reporting no new scientific information on the metabolic phenotypes whatsoever. Its relevance relay on the fact that is methodologically correct study of a Latin American population, which is largely underrepresented in the international literature.

 Nevertheless, there are several specific caveats that must be addressed before the article be suitable for indexing:

Abstract:

 Methodololgy:

1. It must tell the source of the sample to make clear that is a city-wide health survey.
2. It must indicate what was the specific criteria for this clustering individuals in healthy/unhealthy categories. It is not enough to tell that is was made upon a 2-step clustering analysis.

Results:

1. It should be re written to clearly indicate the frequency of each phenotype category and indicate if there were differences between the sexes in this distribution and the OR for casrdiovascular risk factors or diseases.
2. It must indicate what is the difference between unhealthy lean and unhealthy normal-weight individuals

Conclusions: There are no reasons to suppose that individuals of Maracaibo city will have no “atypical metabolic phenotypes” as the rest of the world populations, thus the authors should rephrase this sentence to make more scientifically sound. Also, the conclusion relative to the increased cardiovascular risk of “healthy obese” individuals should be better explained since it cannot be derived from the data reported in the

 Results section of the abstract.

Introduction:

 The phrase: “For this reason, the actual clinical practice catalogues an obese patient as an “unhealthy” patient and a lean patient is considered “healthy”, should be modified to make its medical meaning clearer, because it is evident for everybody that many lean people are unhealthy. 

 Also, the paper will gain interest if the authors comment what is the importance of researching the “atypical phenotypes”in general. For example, is there any evidence that these individuals can be misclassified in their cardiometabolic risk based solely in the BMI? 

 Finally, it is important that the authors comment the extent to what Maracaibo city population is representative of other Latin American populations. For international readers will be interesting to learn that American populations are extremely heterogenous in both genetic and cultural aspects. 

Materials and methods:

 “Population selection”: this whole methodological section is cryptic and is basically a summary of the published in the reference 9. I suggest to re write it to make more understandable for general readers. Specifically, it must be justified why the authors did choose not to use more a conventional definition of metabolic health, such as the metabolic syndrome definition used by ATP III guidelines. 

 “Physical activity”: it should be improved the explanation of what is the relevance and connection of table 1 with the rest of the paper. Also, in this table there is no quantitative definition of the “Work domain” and “lower/upper limit” categories. Information of the proportion of each category over the overall would be useful to summarize these data.

 “Calibration of the Framingham-Wilson equation and coronary risk categorization for the population of Maracaibo city”: the authors must explain what asymptomatic angina is, since medically angina is a symptom by itself.

Results:

 Table 2: change “Indian-american” for “native American”, if its corresponds since “Indian” correspond to India nationals

 “Metabolic phenotypes and biologic-anthropometric variables” section: Since all the variables in this table are statistically different, a post test comparing individual groups should be important to make sense of the noted global differences. 

 Also, a better description and explanation of these particular results is required in the main text.

 “Metabolic phenotypes and coronary risk classification” section: the meaning of the first phrase must be clarified since only obese individuals showed increased OR: other comparisons were not statistically significant with the adjusted model, thus were not different.

Is the work clearly and accurately presented and does it cite the current literature?

Partly

If applicable, is the statistical analysis and its interpretation appropriate?

I cannot comment. A qualified statistician is required.

Are all the source data underlying the results available to ensure full reproducibility?

Yes

Is the study design appropriate and is the work technically sound?

Yes

Are the conclusions drawn adequately supported by the results?

Yes

Are sufficient details of methods and analysis provided to allow replication by others?

Partly

Reviewer Expertise:

Basic and clinical research on adipose tissue disorders, diabetes, dyslipidemia and fatty liver

I confirm that I have read this submission and believe that I have an appropriate level of expertise to confirm that it is of an acceptable scientific standard, however I have significant reservations, as outlined above.
